# Unplanned-start peritoneal dialysis in Brazil: great results, little application

**DOI:** 10.1590/2175-8239-JBN-2023-E002en

**Published:** 2023-03-13

**Authors:** Viviane Calice-Silva, Fabiana Baggio Nerbass

**Affiliations:** 1 Fundação Pró-Rim, Joinville, SC, Brazil; 2 Universidade da Região de Joinville, Joinville, SC, Brazil

Unplanned peritoneal dialysis (PD) initiation, also known as urgent-start PD, was initially defined by the International Society of Peritoneal Dialysis (ISPD) as when therapy is initiated within 14 days of peritoneal catheter insertion^1^. More recently, Blake and Jain proposed that the term urgent start PD should only be used for cases in which there is genuine clinical urgency to start therapy within 72 hours after Tenckhoff catheter insertion^2^. Different cut-off points for treatment initiation have been used in studies comparing outcomes of patients with unplanned or planned treatment initiation. However, the results have been shown to be similar regardless of this fact.

A recent systematic review and meta-analysis evaluating the feasibility and safety of unplanned PD found no difference in mortality, peritonitis, exit site infection, or PD technique survival compared with planned PD. However, a higher incidence of catheter leakage and mechanical dysfunction was observed in the unplanned initiation group^3^. These findings are not different from the still few Brazilian publications on the topic^4,5^, and are now corroborated by Muller and Ponce in a retrospective cohort analysis with more than three hundred patients^6^. The authors compared different outcomes of 206 patients who started PD up to 72 hours (unplanned) after catheter implantation with those of 99 patients whose therapy started after seven days of insertion (defined by the authors as planned) between 2014 and 2020. Among the main results, it was found that technique and patient survival was similar in both groups and that leakage was more frequent with unplanned PD initiation^6^.

Another unique contribution of the work by Muller and Ponce is the identification of etiological causes of infection, allowing the determination of their patients’ microbiota and increasing the chance of treatment success, which impacts technique and patient survival^6^. Given the impact of microbial identification on patient outcomes and treatment success, the updated ISPD peritonitis guideline recommends a negative culture percentage of less than 15% yearly, which is a challenge to many PD services in our country due to the lack of laboratories specialized in those procedures^7^.

Infections and mechanical complications are the two main reasons for technique failure and patient dropout in PD. Although Muller and Ponce’s study demonstrated that leakage is more common with unplanned PD initiation, this is not an indicator of PD failure or patient dropout. There were no predictors for technique failure and mechanical complications identified in this study, and diabetes was the only predictor of peritonitis events (HR 2.02, 95%CI: 1.25-3.25; P= 0.04). Predictors of death were older age and lower albumin levels. Despite such encouraging results, PD is little used as renal replacement therapy (RRT) in our country. According to the last Brazilian Dialysis Survey, although 48% of the 252 participating centers offer PD as an option for RRT, only 5.8% of dialysis patients were treated by this modality. This percentage is decreasing over the years^8^.

Therefore, evaluating why PD has been underutilized in our country is important. Considering the lack of HD places in some regions and the territorial extent of the country, PD could be a safe option for the treatment of end-stage kidney disease (ESKD) in an urgent situation when there is a lack of time to prepare for dialysis initiation. Unfortunately, this is a widespread situation faced in our daily clinical practice due to undiagnosed CKD or late diagnosis. In addition, there are some known barriers, such as difficulties in peritoneal dialysis catheter placement, lower reimbursement compared to hemodialysis, lack of expertise of nephrologists in prescribing and dealing with PD complications, high costs for delivery of supplies, and lack of patient knowledge about this therapy and its advantages.

Considering the increasing number of patients in need of RRT in Brazil and the high economical burden of hemodialysis on the health care system, stakeholders and the government must work together to develop strategies to overcome the above barriers and improve patient care. Implementing an unplanned PD program is an excellent strategy to increase the diffusion of PD, especially by optimizing the utilization of this RRT modality in our country, allowing more people to receive treatment and improving patient outcomes.
